# Joint Optimization of Codeword Bit Distribution and Detection Threshold for Asymmetric STT-MRAM Channel

**DOI:** 10.3390/s26051442

**Published:** 2026-02-25

**Authors:** Thien An Nguyen, Jaejin Lee

**Affiliations:** Department of Information Communication Convergence Technology, Soongsil University, Seoul 06978, Republic of Korea; anthienng1995@soongsil.ac.kr

**Keywords:** asymmetric write error rate, cascaded channel, codeword bit distribution, detection threshold optimization, sparse modulation, error correction codes (ECCs), spin-transfer torque magnetic random-access memory (STT-MRAM), non-volatile memory reliability

## Abstract

Asymmetric error characteristics in spin-transfer torque magnetic random-access memory (STT-MRAM), particularly the imbalance between logical ‘0’ and ‘1’ error probabilities, can significantly degrade system reliability under conventional modulation and error-correcting schemes. This issue is especially critical in sensor network applications, where STT-MRAM is widely adopted for its non-volatility, low standby power, and robustness under energy-constrained and intermittently active operation. Existing approaches typically optimize the detection threshold under the assumption of a fixed or equiprobable bit distribution, while sparse coding techniques impose a predefined imbalance without explicitly accounting for its interaction with threshold detection. In this paper, we formulate the bit error rate (BER) minimization problem as a joint optimization of the codeword bit distribution and the detection threshold over an asymmetric cascaded STT-MRAM channel. Analytical results reveal that the minimum BER is achieved when the error probabilities associated with transmitted ‘0’ and ‘1’ bits are balanced, which induces an intrinsic coupling between the optimal detection threshold and the codeword composition. Motivated by this insight, we propose a new family of threshold-matched probability codes (TMPCs), in which the proportion of logical ‘1’s in each codeword is explicitly designed to match the optimal detection threshold of the underlying channel. The proposed coding framework generalizes conventional sparse modulation by enabling adjustable bit distributions while preserving low-complexity linear encoding and syndrome-based decoding. Numerical evaluations demonstrate that the TMPC achieves consistently lower BERs than existing sparse and fixed-distribution coding schemes across a wide range of STT-MRAM operating conditions, particularly under severe write asymmetry and resistance variation. These results indicate that the proposed joint design offers a principled and flexible approach for improving reliability in STT-MRAM-based sensor networks and non-volatile memory systems.

## 1. Introduction

In modern sensor-centric systems, memory is no longer a passive storage component but a critical subsystem that directly influences sensing reliability, energy efficiency, and overall system lifetime. Smart sensors continuously interact with the physical environment and must frequently store raw sensing data, intermediate processing results, and system states. This operational paradigm imposes stringent requirements on memory technologies, including high write endurance, low access latency, energy efficiency, and robust data reliability [1]. In battery-powered and energy-harvesting sensor nodes, inefficient or unreliable memory operation can severely degrade sensing accuracy and significantly shorten system lifetime, making memory design a key determinant of sensor network performance.

Conventional volatile memories, such as dynamic random-access memory (DRAM) and synchronous DRAM (SDRAM), are often ill-suited for sensor platforms due to their volatility and high standby power consumption. The need for continuous refresh operations not only wastes energy but also increases system complexity, which is undesirable in resource-constrained sensor nodes and large-scale sensor networks [1]. Consequently, there has been growing interest in non-volatile memory technologies that enable low-power operation, instant-on functionality, and reliable data retention in sensor systems.

Among emerging non-volatile memories, magnetic random-access memory (MRAM) has attracted considerable attention for sensor-oriented and networked sensing applications due to its unique combination of non-volatility, fast access speed, low leakage power, and high endurance. These characteristics are particularly advantageous for sensing data buffering, event-driven storage, and intermittent operation, which are common in wireless sensor networks and Internet of Things (IoT) platforms [2]. As a result, MRAM has been widely regarded as a key enabler of unified embedded non-volatile memory architectures in sensor networks and IoT nodes [3].

Within the MRAM family, spin-transfer torque MRAM (STT-MRAM) is especially attractive for sensor-centric and sensor-networked systems. By combining DRAM-like access speed and endurance with non-volatile data retention, STT-MRAM supports efficient and reliable memory operation under the tight energy budgets, frequent access patterns, and intermittent power conditions characteristic of sensor workloads [4,5]. These attributes position STT-MRAM as a promising memory technology for next-generation sensor networks that demand both high reliability and long operational lifetime.

In STT-MRAM-based sensor systems, digital information is accessed through an nMOS transistor that controls the read and write current, while data are physically stored in the resistance state of a magnetic tunnel junction (MTJ). Each MTJ consists of two ferromagnetic layers separated by an ultrathin tunneling oxide barrier: a fixed reference layer and a switchable free layer. Depending on the relative magnetization orientation of these two layers, the MTJ exhibits either a low-resistance state (parallel alignment) or a high-resistance state (antiparallel alignment), which are interpreted as binary data during sensing and readout operations [6].

Owing to its non-volatility, fast access speed, and high endurance, STT-MRAM has been widely regarded as a strong candidate for embedded non-volatile memory in sensor-oriented platforms and distributed sensor networks [7]. Nevertheless, when deployed in practical sensor systems, the reliability of STT-MRAM can be significantly affected by various device- and circuit-level nonidealities [8]. Among these, process variation plays a dominant role, as inevitable fluctuations in MTJ dimensions and material properties lead to resistance dispersion and variations in switching current thresholds [9]. Such variations directly degrade read margins and sensing accuracy, which are critical for reliable data acquisition in sensor applications.

Timing constraints in synchronous memory architectures introduce additional challenges. Write operations are often confined to predefined time windows, and under process and temperature variations, some MTJs may fail to complete state transitions within the allotted duration [7]. Thermal noise further exacerbates this issue by introducing stochasticity into the switching dynamics. To mitigate temperature-induced instability during read operations, practical STT-MRAM designs frequently employ MTJ-based sensing circuits combined with self-referencing schemes or poly-resistor references [10]. However, these techniques cannot fully suppress resistance fluctuations, which may still induce unintended state changes even in the absence of explicit write commands [11].

Another critical reliability concern in sensor-oriented STT-MRAM is read disturbance, in which the read current itself perturbs the magnetic state of the free layer. This phenomenon can cause both read and write failures, thereby compromising data integrity in memory-intensive sensing workloads. As technology continues to scale and sensing margins shrink, the combined effects of process variation, thermal fluctuation, and read disturbance are expected to intensify. These challenges underscore the need for advanced design methodologies that jointly consider device characteristics, sensing mechanisms, and system-level requirements to enhance the robustness of STT-MRAM in sensor network applications.

A variety of hardware-oriented approaches have been proposed to address these limitations, ranging from circuit-level optimizations to architectural enhancements. Prior studies have shown that optimizing transistor dimensions beyond conventional worst-case sizing can substantially reduce write energy consumption [12]. Integrated magnetic–circuit co-design techniques have also been employed to further improve write efficiency [12]. At the architectural level, Dong et al. proposed a heterogeneous memory system combining static random-access memory (SRAM) with STT-MRAM to balance performance and reliability [13], while the use of write buffers to mitigate write-induced performance degradation was examined in [14]. The impact of manufacturing variation has also been extensively studied: Wang et al. analyzed the role of thermal fluctuations in MTJ devices [15], and Smullen et al. introduced a thermal noise modeling framework to capture resistance variations during the MTJ switching process [16].

Errors occurring during write and read operations can be mitigated through error-correcting codes (ECC). For example, BCH codes with multi-bit error correction capability were presented in [17]. Similar to their use in other communication and storage systems [18,19], low-density parity-check (LDPC) codes have been adopted in STT-MRAM to achieve near-Shannon-limit performance [20]. Adaptive ECC schemes aimed at reducing redundancy overhead were further investigated in [21,22].

Channel modeling has played a central role in the development and evaluation of error-correcting techniques for STT-MRAM [23,24]. In particular, the cascaded channel representation proposed in [25] provides a practical abstraction that enables detailed error-rate analysis by modeling STT-MRAM as a communication channel. Using this framework, Nguyen demonstrated that bit errors in STT-MRAM are statistically independent and exhibit asymmetric transition probabilities between logical states [7]. This asymmetry motivated the 7/9 sparse coding scheme in [7], which biases codewords toward zeros to match dominant error patterns. Nguyen and Lee further improved this approach by enforcing a minimum Hamming distance of three to enhance error resilience [26]. However, the mapping-based encoding and decoding employed in [26] incur considerable computational complexity and memory overhead. To address this limitation, generator-matrix-based constructions were proposed in [27,28] to enable direct encoding and decoding of user data. Nevertheless, these approaches rely on fixed code structures, with the density of logical ‘1’ bits treated as a constant estimated parameter in BER analysis. This assumption restricts the optimization space and limits achievable BER performance.

More recently, several studies have investigated reliability enhancement and sensing optimization in STT-MRAM under process variation and asymmetric switching conditions [29,30,31]. These works primarily focus on device-level modeling, adaptive ECC schemes, or sensing margin improvement. However, they do not explicitly formulate the joint optimization of codeword bit distribution and detection threshold under an analytical BER framework.

In contrast, this work proposes a threshold-matched probability coding (TMPC) framework that fundamentally departs from prior designs. Rather than fixing the code structure in advance, TMPC formulates the BER as a joint function of the detection threshold and the probability of logical ‘1’ bits, treating the bit probability as a primary optimization variable. By first identifying the optimal bit distribution that minimizes the BER under asymmetric STT-MRAM read conditions, TMPC guides the construction of codewords whose compositions are matched to this optimal probability. This optimization-first, code-aware design philosophy enables a more comprehensive exploration of the BER landscape and achieves performance closer to the global optimum than existing mapping-based and generator-matrix-based schemes. Simulation results demonstrate that the proposed TMPC consistently provides significant BER improvements across a wide range of channel conditions.

Although prior works have proposed sparse coding techniques and threshold optimization strategies to mitigate asymmetric write errors in STT-MRAM [7,26,27,28], these approaches typically treat the detection threshold and the bit distribution as independent design variables. Sparse modulation schemes usually adopt a predetermined bit-one density [7,26], while threshold optimization methods assume fixed or equiprobable bit distributions under cascaded channel models [25]. Consequently, the intrinsic coupling between channel asymmetry and codeword composition remains insufficiently explored.

In contrast, this work formulates the BER minimization problem as a joint optimization of both detection threshold and codeword bit distribution under an entropy constraint. By analytically characterizing the interaction between channel asymmetry and bit probability, the proposed TMPC framework achieves performance closer to the theoretical optimum without increasing online encoding or decoding complexity. To the best of our knowledge, such a joint analytical optimization framework has not been explicitly addressed in recent STT-MRAM reliability studies.

The remainder of this paper is organized as follows: Section 2 introduces the STT-MRAM read channel model and formulates the BER minimization problem under asymmetric read conditions. Section 3 presents the proposed TMPC framework, including joint optimization of the codeword bit distribution and detection threshold, as well as the corresponding code construction methodology. Section 4 presents simulation results and performance comparisons with existing mapping-based and generator-matrix-based schemes. Finally, Section 5 concludes the paper and discusses directions for future research.

## 2. Channel Model

**Figure 1:**
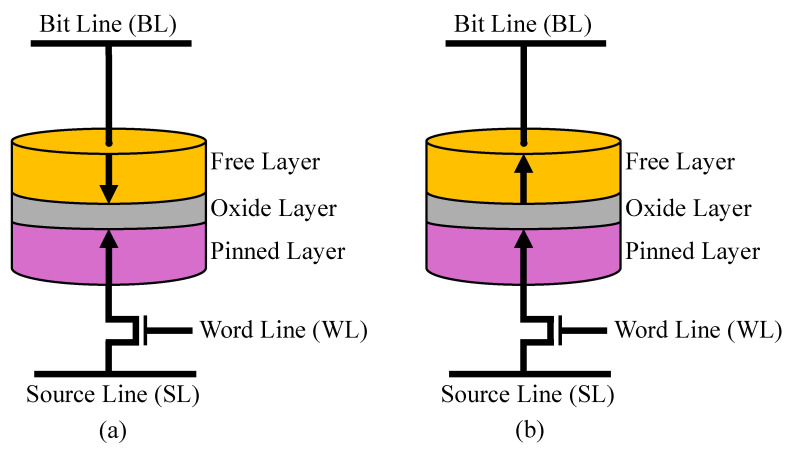
Schematic illustration of an STT-MRAM cell operating in (**a**) the antiparallel state (AS) and (**b**) the parallel state (PS).

Figure 1 illustrates the basic configuration of an STT-MRAM cell. Each cell adopts a one-transistor–one-MTJ (1T1J) structure, in which an nMOS access transistor is connected in series with a magnetic tunnel junction (MTJ). The access transistor controls the current path during read and write operations, while the MTJ serves as the non-volatile storage element.

The MTJ consists of two ferromagnetic layers separated by an ultrathin insulating barrier. One layer, referred to as the reference layer, has a fixed magnetization orientation, whereas the other layer, known as the free layer, can switch its magnetization under the influence of a spin-polarized current. Information is stored according to the relative alignment of the magnetizations in these two layers. When the magnetizations are aligned in parallel (P), the MTJ exhibits a low-resistance state, denoted as $R_{P}$. Conversely, an antiparallel (AP) alignment results in a high-resistance state, denoted as $R_{AP}$. In this work, the parallel and antiparallel states correspond to logical ‘0’ and logical ‘1’, respectively.

Data retrieval in STT-MRAM is performed by sensing the MTJ resistance and comparing it with a predefined reference threshold. Owing to device-level variations, noise, and environmental fluctuations, the resistance values associated with the parallel and antiparallel states are not deterministic but instead follow statistical distributions that may partially overlap. Consequently, the choice of the sensing threshold critically affects read accuracy. This resistance-based sensing behavior forms the foundation of the read channel model adopted in this study and motivates the optimization strategies presented in subsequent sections.

The STT-MRAM read process can be abstracted as a communication system, in which the stored magnetic state corresponds to the transmitted symbol and the sensed resistance represents the received signal. This cascaded channel abstraction enables sensing reliability to be analyzed using communication-theoretic tools.

Programming a logical ‘0’, corresponding to the parallel state (PS), is achieved by driving a current from the free layer toward the reference layer. In this write-0 configuration, the supply voltage $V_{DD}$ is applied to the bit line (BL) and the word line (WL), while the source line (SL) is connected to ground. The resulting current induces spin-transfer torque that aligns the free-layer magnetization with that of the reference layer. In contrast, writing a logical ‘1’ sets the MTJ to the antiparallel state (AS) by reversing the current direction. In this write-1 mode, $V_{DD}$ is applied to the source line (SL) and the word line (WL), whereas the bit line (BL) is grounded. The resulting current flows from the reference layer to the free layer, causing the free-layer magnetization to switch to the antiparallel orientation.

Read operations are performed by injecting low-magnitude sensing current through the MTJ. A dedicated sensing circuit measures the resulting voltage or resistance and determines the stored data by comparing the measured resistance with a reference threshold. A high resistance indicates the AP state, whereas a low resistance corresponds to the P state. Although the read current polarity is identical to that used in the corresponding write operation, its amplitude is carefully limited to prevent unintended magnetization switching. Therefore, accurate resistance sensing and proper threshold selection are essential to ensure non-destructive readout.

The reliability of STT-MRAM cells is strongly influenced by fabrication-induced process variations and thermal noise, both of which contribute to write and read failures. Write failures occur when the applied switching current fails to generate sufficient spin-transfer torque to deterministically reverse the MTJ magnetization. In such cases, the MTJ remains in its original state instead of completing the intended transition, whether from logical ‘1’ to ‘0’ (antiparallel to parallel) or from logical ‘0’ to ‘1’ (parallel to antiparallel).

Among these transitions, the 0 → 1 switching process is particularly vulnerable, as it requires a higher critical current due to its lower intrinsic STT efficiency compared with the reverse transition. As a result, the write error probability associated with the 0 → 1 transition (*P*_1_) is significantly higher than that of the 1 → 0 transition (*P*_0_), a phenomenon consistently reported in prior studies [32,33,34]. Specifically, failures during the parallel-to-antiparallel (P → AP) transition occur with much higher probability than those during the antiparallel-to-parallel (AP → P) direction. Thermal agitation further degrades write reliability by introducing stochasticity into the magnetization dynamics, thereby increasing switching variability and exacerbating data integrity concerns.

Read failures in STT-MRAM can be broadly classified into two categories: sensing errors and read-disturbance-induced errors. Sensing errors arise when the read circuit fails to reliably distinguish between MTJ resistance states, often due to limited sensing margins or resistance fluctuations, leading to incorrect bit decisions. In contrast, read disturbance errors occur when excessive read currents unintentionally perturb or even flip the magnetic state of the MTJ during the read operation.

The effective application of signal processing techniques to STT-MRAM relies on an accurate channel representation that captures both the physical characteristics of the device and the dominant sources of performance degradation. In this work, we adopt the cascaded channel model originally proposed by Cai and Immink [25], which provides a unified framework for modeling the combined effects of write failures and read imperfections under realistic operating conditions. A concise description of this model is provided below.

Let *P*_1_ and *P*_0_ denote the write error probabilities associated with the 0 → 1 and 1 → 0 switching events, respectively. In the cascaded channel formulation, write and read error mechanisms are jointly considered. Write failures are modeled using a binary asymmetric channel (BAC), in which the transition probabilities for 0 → 1 and 1 → 0 errors are given by *P*_1_/2 and *P*_0_/2, respectively.

Read-related failures are further decomposed into read disturbance errors and sensing decision errors. Read disturbance effects are modeled using a Z-channel, where *P*_r_ denotes the probability of an unintended state transition induced by the read operation, depending on whether a logical ‘0’ or ‘1’ was previously written. Sensing decision errors are characterized using a Gaussian mixture channel (GMC), in which the MTJ resistance values are modeled as Gaussian random variables: a low-resistance state *R*_0_ with mean *μ*_0_ and variance *σ*_0_^2^ and a high-resistance state *R*_1_ with mean *μ*_1_ and variance *σ*_1_^2^.

**Figure 2:**
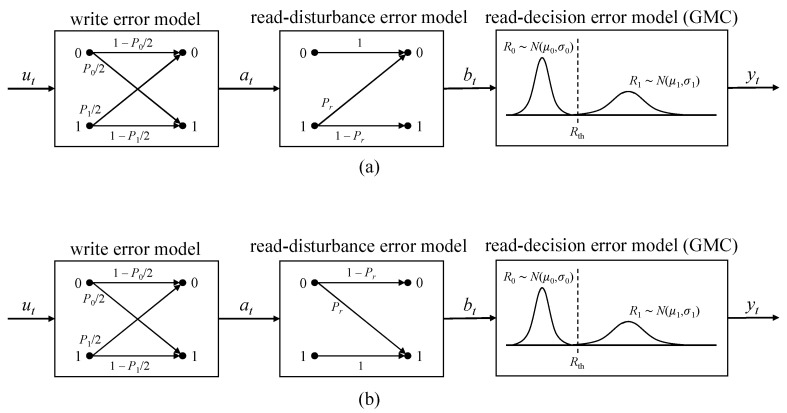
Cascaded channel model. (**a**) Reading with a write-0 direction. (**b**) Reading with a write-1 direction.

By cascading the BAC-based write error model, the Z-channel representation of read disturbances, and the GMC-based sensing error model, a comprehensive end-to-end channel representation for STT-MRAM is obtained. The overall structure of the cascaded channel model is illustrated in Figure 2.

The cascaded write–read channel model adopted in this work follows the widely used statistical abstraction in STT-MRAM reliability analysis. Recent studies have further investigated high-speed sensing circuits and dynamic time-domain sensing schemes to improve read stability under process and thermal variations [29,30]. In parallel, coding techniques for resistive memory systems have been explored to mitigate asymmetric error characteristics [31]. These studies confirm that asymmetric write behavior and sensing variation remain dominant reliability-limiting factors in practical STT-MRAM systems.

The adopted cascaded channel model captures these two effects within a unified analytical framework, thereby providing a tractable yet representative basis for subsequent BER optimization.

**Figure 3:**
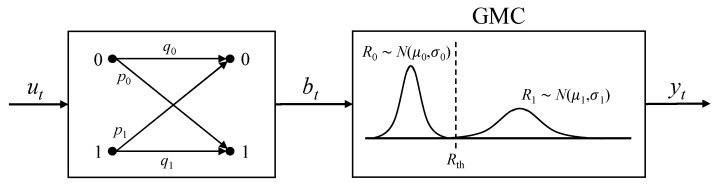
Cascaded channel combination model.

Furthermore, the write error mechanism captured by the BAC and the read disturbance effect can be jointly represented by an equivalent simplified channel, as shown in Figure 3. Under this combined formulation, the effective crossover probabilities of the resulting channel are derived as follows:

For the write-0 direction, the transition probabilities can be expressed as:

(1) $$\begin{array}{l} p_{0}=\frac{P_{0}}{2}\left(1-P_{r}\right);\;q_{0}=\left(1-\frac{P_{0}}{2}\right)+\frac{P_{0}}{2}P_{r};\\ p_{1}=\frac{P_{1}}{2}+\left(1-\frac{P_{1}}{2}\right)P_{r};\;q_{1}=\left(1-\frac{P_{1}}{2}\right)\left(1-P_{r}\right). \end{array}$$

Similarly, for the write-1 direction, the transition probabilities are given by:

(2) $$\begin{array}{l} p_{0}=\frac{P_{0}}{2}+\left(1-\frac{P_{0}}{2}\right)P_{r};\;q_{0}=\left(1-\frac{P_{0}}{2}\right)\left(1-P_{r}\right);\\ p_{1}=\frac{P_{1}}{2}\left(1-P_{r}\right);\;q_{1}=\left(1-\frac{P_{1}}{2}\right)+\frac{P_{1}}{2}P_{r}. \end{array}$$

Under the above cascaded channel model, the overall BER depends jointly on the detection threshold and the codeword bit distribution. However, conventional approaches typically optimize these parameters independently. This observation motivates the joint analytical optimization framework proposed in the next section.

## 3. Proposed Joint Optimization Model

In conventional STT-MRAM reliability design, the detection threshold and the bit distribution are typically determined independently. The detection threshold is selected based on sensing statistics under the assumption of equiprobable input bits, while the bit distribution is fixed by the coding scheme. Under this approach, the BER is evaluated with predetermined parameters rather than jointly optimized.

However, as will be shown in the following derivation, the BER under the cascaded channel model depends jointly on both the detection threshold and the bit distribution. Optimizing only one parameter while keeping the other fixed may lead to suboptimal performance under asymmetric channel conditions.

Therefore, we formulate BER minimization as a joint optimization problem over both the detection threshold and the codeword bit distribution under an entropy constraint.

The threshold update step is closely related to the classical likelihood ratio test (LRT), which minimizes detection error for fixed prior probabilities. When the bit distribution is equiprobable, the optimal threshold reduces to the conventional LRT solution. However, unlike standard detection theory where prior probabilities are assumed fixed, the proposed framework treats the bit distribution as an additional optimization variable under an entropy constraint. This joint treatment enables further BER reduction under asymmetric channel conditions and extends beyond traditional likelihood-based threshold optimization.

### 3.1. Asymmetric STT-MRAM Read Channel Model

During read operations, the resistance of an MTJ cell is sensed and compared with a reference threshold *R_th_*. Due to process variation, thermal noise, and device nonidealities, the resistance values corresponding to the P and AP states are modeled as Gaussian random variables:

(3) $$R_{0}∼N\left(\mu_{0},\sigma_{0}^{2}\right),$$

(4) $$R_{1}∼N\left(\mu_{1},\sigma_{1}^{2}\right),$$

where *μ*_0_, *σ*_0_, and *μ*_1_, *σ*_1_ denote the mean and standard deviation of the resistance distributions associated with the P and AP states, respectively.

A hard-decision read operation is performed as

(5) $$\hat{x}=\left\{\begin{array}{cc} 0, & R\leq R_{th},\\ 1, & R>R_{th}. \end{array}\right.$$

where $\hat{x}$ denote the detected bit. Let *P*_0_ and *P*_1_ = 1 − *P*_0_ denote the prior probabilities of transmitting logical ‘0’ and ‘1’, respectively.

### 3.2. BER Formulation as a Function of R_th_ and Bit Distribution

Under the cascaded asymmetric read channel depicted in Section 2, the overall bit error rate (BER) can be expressed as

(6) $$\begin{array}{l} P_{b}(P_{0},R_{th})=P_{0}\left[q_{0}Q\left(\frac{R_{th}-\mu_{0}}{\sigma_{0}}\right)+p_{0}Q\left(\frac{R_{th}-\mu_{1}}{\sigma_{1}}\right)\right]\\ \;\;\;\;\;\;\;\;\;\;\;\;\;\;\;\;\;\;\;\;\;\;+P_{1}\left[q_{1}\left(1-Q\left(\frac{R_{th}-\mu_{1}}{\sigma_{1}}\right)\right)+p_{1}\left(1-Q\left(\frac{R_{th}-\mu_{0}}{\sigma_{0}}\right)\right)\right], \end{array}$$

where *Q*(⋅) denotes the Gaussian Q-function, and *p_i_*, *q_i_* represent the crossover probabilities of the cascaded channel for the corresponding bit values.

Equation (6) explicitly shows that the BER depends not only on the sensing threshold *R_th_* but also on the prior bit distribution (*P*_0_, *P*_1_), which is determined by the codeword composition. In sensor systems, such read errors directly manifest as corrupted sensing data or erroneous intermediate results, which may propagate through subsequent signal processing stages and degrade overall sensing accuracy and system reliability.

### 3.3. Entropy Constraint for Degeneracy Prevention

Direct minimization of the BER in (6) may lead to degenerate solutions in which the bit distribution collapses to all zeros or all ones. To prevent such trivial solutions, an entropy constraint is imposed.

The binary entropy associated with $P_{0}$ is given by:

(7) $$H\left(P_{0}\right)=-P_{0}{\;\log}_{2}\;P_{0}-\left(1-P_{0}\right)\log_{2}\left(1-P_{0}\right),$$

which satisfies $0\leq H(P_{0})\leq 1$, with the maximum achieved at $P_{0}=0.5$.

Rather than enforcing maximum entropy, which would preclude exploitation of channel asymmetry, we constrain the entropy to lie within a predefined range:

(8) $$H_{\min}\leq H\left(P_{0}\right)\leq H_{\max},$$

where $0<H_{min}<H_{max}<1$. This constraint preserves sufficient information content while allowing a controlled bias toward the more reliable resistance state.

### 3.4. Joint Optimization Problem Formulation

The proposed framework jointly optimizes the detection threshold and the bit distribution to minimize the BER:

(9) $$\begin{array}{l} \underset{R_{th},P_{0}}{\min}\;P_{b}\left(P_{0},R_{th}\right)\\ \;s.t.\;0\leq P_{0}\leq 1,\\ \;\;\;\;\;\;\;\;\;P_{1}=1-P_{0},\\ \;\;\;\;\;\;\;\;\;\mu_{0}\leq R_{th}\leq \mu_{1},\\ \;\;\;\;\;\;\;\;\;H_{\min}\leq H\left(P_{0}\right)\leq H_{\max} \end{array}$$

Here, the BER serves as the primary optimization objective, while the entropy constraint acts as a regularization mechanism that eliminates degenerate solutions with vanishing information content.

When $H_{max}=1$ (i.e., $P_{0}=0.5$), the problem reduces to threshold-only optimization. Although this approach partially exploits channel asymmetry at the detection level, it fails to leverage distribution-level asymmetry and therefore yields suboptimal BER performance.

### 3.5. Alternating Optimization of R_th_ and P_0_

For a fixed bit distribution *P*_0_, the optimal threshold $R_{th}^{*}$ is obtained by solving

(10) $$\frac{\partial P_{b}\left(P_{0},R_{th}\right)}{\partial R_{th}}=0$$

This leads to a nonlinear equation involving exponential and Q-function terms, which can be efficiently solved using numerical methods such as the Newton–Raphson or bisection algorithm. The resulting optimal threshold can be expressed as

(11) $$R_{th}^{*}=f\left(P_{0}\right)$$

indicating that the optimal detection threshold explicitly depends on the underlying bit distribution.

Conversely, for a fixed threshold *R_th_*, the BER is linear in $P_{0}$:

(12) $$P_{b}=P_{0}E_{0}\left(R_{th}\right)+\left(1-P_{0}\right)E_{1}\left(R_{th}\right),$$

where

(13) $$E_{0}\left(R_{th}\right)=P_{0}\left[q_{0}Q\left(\frac{R_{th}-\mu_{0}}{\sigma_{0}}\right)+p_{0}Q\left(\frac{R_{th}-\mu_{1}}{\sigma_{1}}\right)\right]$$

(14) $$E_{1}\left(R_{th}\right)=P_{1}\left[q_{1}\left(1-Q\left(\frac{R_{th}-\mu_{1}}{\sigma_{1}}\right)\right)+p_{1}\left(1-Q\left(\frac{R_{th}-\mu_{0}}{\sigma_{0}}\right)\right)\right]$$

The unconstrained optimum satisfies

(15) $$E_{0}\left(R_{th}^{*}\right)=E_{1}\left(R_{th}^{*}\right)$$

Without constraints, this leads to $P_{0}\in \{0,1\}$, i.e., degeneracy. Under the entropy constraint in (8), the feasible region becomes bounded.

(16) $$P_{0}\in \left[P_{0}^{\min},P_{0}^{\max}\right]$$

Since (12) is linear in *P*_0_, the constrained optimum lies on one of the entropy boundaries.

The joint optimization is solved using an alternating minimization scheme, as summarized in Algorithm 1.
**Algorithm 1.**$\;\mathrm{Alternating}\;\mathrm{Optimization}\;\mathrm{of}\;R_{\mathrm{th}}\;\mathrm{and}\;P_{0}$**Input:** Channel parameters (*μ*_0_, *μ*_1_, *σ*, *P_r_*, *P*_1_$),\;\mathrm{initial}\;\mathrm{bit}\;\mathrm{probability}\;P_{0}^{\left(0\right)}$, entropy constraint *H*_min_, tolerance *ε*, maximum iteration number *I*_max_. **Output:**
$\mathrm{Optimized}\;\mathrm{detection}\;\mathrm{threshold}\;R_{th}^{*}$$\;\mathrm{and}\;\mathrm{optimized}\;\mathrm{bit}\;\mathrm{probability}\;P_{0}^{*}$.$\mathrm{Initialize}\;P_{0}^{\left(0\right)}$$\;\mathrm{such}\;\mathrm{that}\;\mathrm{the}\;\mathrm{entropy}\;\mathrm{constraint}\;H_{min}$ is satisfied.Set iteration index k=0Repeat:
$\mathrm{Update}\;R_{th}^{\left(k+1\right)}$$\;\mathrm{for}\;\mathrm{fixed}\;P_{0}^{\left(k\right)}$.$\mathrm{Update}\;P_{0}^{\left(k+1\right)}$$\;\mathrm{for}\;\mathrm{fixed}\;R_{th}^{\left(k+1\right)}$ under the entropy constraint.Compute the corresponding BER value.Set k=k+1.The iteration stops when the convergence condition is satisfied.$\mathrm{Return}\;R_{th}^{*}$$\;\mathrm{and}\;P_{0}^{*}$

The alternating optimization terminates when the change in BER between two successive iterations becomes sufficiently small, i.e., $|{\mathrm{BER}}^{\left(k+1\right)}-{\mathrm{BER}}^{\left(k\right)}|\;<\varepsilon ,$ or when the maximum iteration number $I_{max}$ is reached.

In our simulations, we set $\varepsilon =10^{-6}$ and $I_{max}=20$, which were sufficient to ensure convergence.

Sensitivity to model mismatch: Although $\left(P_{1}^{*},R_{th}^{*}\right)$ is obtained under nominal channel parameters, small deviations in sensing statistics or crossover probabilities only introduce a limited BER penalty because the design corresponds to a stationary point of the BER objective with respect to the optimized variables. In addition, TMPC requires no additional online complexity: parameter re-estimation and re-optimization can be performed offline when operating conditions change (e.g., temperature/aging), while the encoder/decoder remains unchanged.

#### Computational Complexity Analysis

The proposed alternating optimization involves two scalar decision variables: the detection threshold $R_{th}$ and the bit distribution parameter $P_{0}$. For a fixed $P_{0}$, updating $R_{th}$ requires solving a one-dimensional nonlinear equation. When implemented using the bisection method, the complexity is O(log(1/ε)), where ε denotes the numerical tolerance. Alternatively, Newton–Raphson typically converges in O(log log(1/ε)) iterations under regular conditions.

For a fixed threshold, the BER expression is linear in $P_{0}$, and the optimal value under the entropy constraint is obtained by evaluating the objective at the boundary points of the feasible interval. This step has constant complexity O(1). 

If the alternating optimization runs for I outer iterations, the total computational complexity is therefore O(I T), where T denotes the number of iterations required by the numerical solver. Since the optimization operates only on scalar channel parameters, its complexity is independent of the codeword length n. Moreover, the optimization is performed offline and does not affect the online encoding or decoding complexity.

### 3.6. Mapping the Optimal Distribution to Code Design (TMPC)

**Figure 4:**
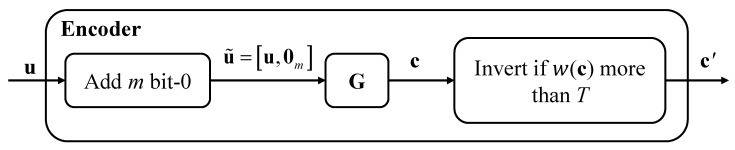
Encoder diagram of STT-MRAM.

Once the optimal bit probability $P_{1}^{*}=1-P_{0}^{*}$ is obtained, it is used to guide the construction of TMPC. The encoder enforces the codeword composition such that

(17) $$\frac{\omega \left(c\right)}{n}\approx P_{1}^{*}$$

where *ω*(*c*) denotes the Hamming weight of codeword ***c*** and *n* is the codeword length. The encoder structure, adapted from [28], is illustrated in Figure 4.

Let *k* denote the number of information bits and *n* denote the codeword length of the linear block code. Let *m* represent the number of appended zero bits. The user data vector is defined as $u\in \{0,1{\}}^{k}$. An extended input vector is formed by appending *m* zeros:

(18) $$\tilde{u}=\left[u,0_{m}\right]\in {\left\{0,1\right\}}^{k+m}$$

where $0_{m}$ denotes the all-zero vector of length *m*. The generator matrix $G\in \{0,1{\}}^{(k+m)\times n}$ corresponds to an (n,k+m) linear block code. The encoded codeword is given by

(19) $$c=\tilde{u}G\;(\mathrm{over}\;\mathrm{GF}(2)).$$

where **c** $\in \{0,1{\}}^{n}$.

The Hamming weight of c is defined as

(20) $$w=w\left(c\right)=\sum_{j=1}^{n}c_{j}.$$

To enforce a low proportion of ones, a conditional bitwise complement operation is applied using a threshold T:

(21) $$c^{\prime }=\left\{\begin{array}{cc} c, & \mathrm{if}\;w\left(c\right)\leq T,\\ 1⊕c, & \mathrm{if}\;w\left(c\right)>T, \end{array}\right..$$

where ⊕ denotes XOR and 1 is the all-one vector of length n. After inversion, the new weight becomes

(22) $$w^{\prime }=w\left(c^{\prime }\right)=\min\left(w,n-w\right).$$

The bit-one probability of the output codeword is defined as the expected fraction of ones:

(23) $$P_{1}≜\mathrm{Pr}\left({c^{\prime }}_{j}=1\right)\approx E\left(\frac{w^{\prime }}{n}\right)$$

where *E*(.) is expectation. This relation holds because $w^{\prime }$ counts the number of ones in $c^{\prime }$, and $\frac{w^{\prime }}{n}$ is the empirical fraction of ones. Let P(w)=Pr(w(c)=w) denote the probability mass function (pmf) of the pre-inversion weight. Then,

(24) $$P_{1}=\sum_{w=0}^{n}\frac{\min\left(w,n-w\right)}{n}P\left(w\right)$$

For many linear encoders with independent and identically distributed. Bernoulli (0.5) inputs, the pre-inversion weight can be approximated by a binomial distribution:

(25) $$\omega \left(c\right)\approx Binomial\left(n,0.5\right),\;P\left(w\right)\approx \left(\begin{array}{c} n\\ w \end{array}\right)2^{-n}$$

Substituting this probability mass function into the boxed equation above yields a numerically computable estimate of $P_{1}$. For large n, a normal approximation w∼N(n/2, n/4) may be used. Under this approximation, selecting

(26) T=τn

typically yields

(27) $$P_{1}\approx \tau$$

providing a simple guideline for selecting the threshold to achieve a target $P_{1}$. Appending m zeros restrict the set of possible codewords (i.e., it selects a subcode), which mainly affects the variance/stability of the weight distribution P(w). However, the target value of $P_{1}$ is primarily determined by the inversion threshold T, while m influences how tightly the realized bit-one probability concentrates around the target. In practice, the target bit-one probability $P_{1}=\tau$ can be achieved by setting the inversion threshold to T=τn, while the zero-appending parameter m is used to control the stability (variance) of the resulting weight distribution.

## 4. Experiments and Results

**Figure 5:**
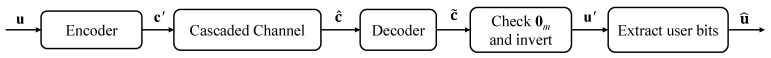
Overall diagram of STT-MRAM system.

The overall STT-MRAM-based sensing and storage system considered in this study is illustrated in Figure 5. In the simulations, the same channel parameters reported in [32] are adopted to ensure a fair comparison with existing schemes. Specifically, the write error probability for the 0 → 1 transition is set to $P_{1}=2\times 10^{-4}$, while the write error probability $P_{0}$ and the read disturbance probability $P_{r}$ are assumed to be two orders of magnitude smaller than $P_{1}$. As a result, the effective crossover probabilities of the cascaded channel become $p_{1}=1.02\times 10^{-4}$ and $p_{0}=10^{-6}$.

To evaluate the BER performance, a user bit sequence **u** of length 1,440,000 bits is generated. This sequence is encoded into a codeword $c^{\prime }$ using a sparse code with a minimum Hamming distance of three. The probability of logical ‘1’ bits in $c^{\prime }$ is adjusted according to the optimized value $P_{1}^{*}$ obtained in Section 3.

The codeword $c^{\prime }$ is then transmitted through the cascaded STT-MRAM channel, yielding the received sequence $\hat{c}$. At the receiver, decoding follows the procedure described in [28] to obtain an intermediate decoded sequence $\tilde{c}$. Based on $\tilde{c}$, the appended zero bits are examined and removed, and a conditional bitwise inversion is applied, if required, to recover the estimated extended data sequence $u^{\prime }$.

Finally, the estimated user data $\hat{u}$ is extracted from $u^{\prime }$ and compared with the original user data **u** to compute the BER. This process is repeated for different threshold and bit-distribution settings to assess the effectiveness of the proposed TMPC scheme.

We perform Monte Carlo simulations in MATLAB R2023b to evaluate the BER performance of the proposed TMPC framework. The cascaded STT-MRAM channel model described in Section 2 is adopted.

Unless otherwise specified, the following parameters are used:

- Write asymmetry probability: *P*_1_ = 2 × 10^−4^.
- Reverse write probability: *P*_0_ and *P_r_* are set two orders of magnitude smaller than *P*_1_.
- Sensing noise modeled as Gaussian with mean *μ*_0_, *μ*_1_ and standard deviation *σ*.
- Code parameters: (*n*, *k*) = (63, 54).
- Entropy constraint *H*_min_ ∈ {0.8, 0.9, 0.99}.
- Number of Monte Carlo trials: 10^6^ bits per operating point.

The detection threshold and bit distribution are optimized according to Algorithm 1 unless otherwise stated.

Although the entropy constraint is defined as $H_{min}\leq H(P_{0})\leq H_{max}$, in practice the BER-driven optimization naturally pushes the solution toward the lower boundary $H(P_{0})=H_{min}$, since lower entropy allows a stronger bias toward the more reliable state and thus results in a lower BER. Consequently, $H_{min}$ primarily determines the operating point in simulations, whereas $H_{max}$ mainly ensures feasibility and prevents trivial configurations. In this work, $H_{max}=0.99$ is chosen as a sufficiently loose upper bound that does not restrict the optimizer while excluding the fully uniform case (H=1), which cannot exploit channel asymmetry.

The entropy constraints $H_{min}=0.8,\;0.95,\;and\;0.99$ are selected to represent different reliability–entropy trade-offs. Extremely low entropy values are excluded because they would imply impractically strong bit bias and reduced effective information content in sensor-network applications.

**Figure 6:**
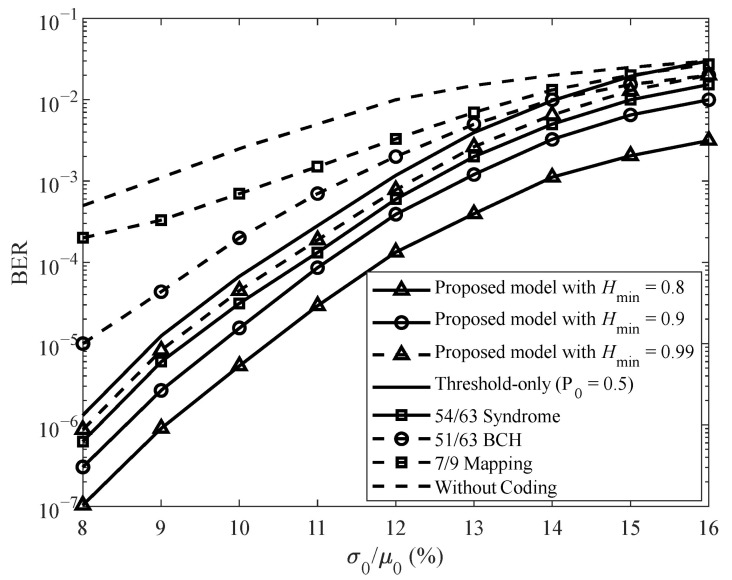
BER performance versus sensing noise $\sigma_{0}/\mu_{0}$ for the proposed TMPC scheme under different entropy constraints.

**Figure 7:**
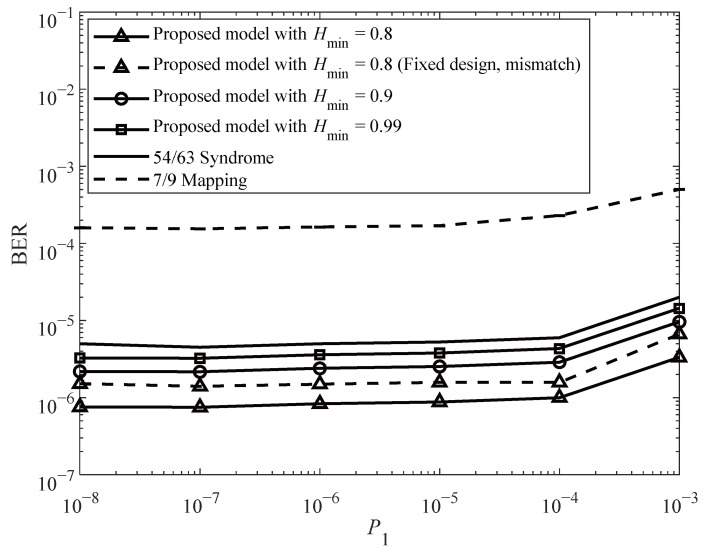
BER performance versus write error rate $P_{1}$.

Sensitivity results in Figure 6 and Figure 7 further show that the proposed TMPC framework maintains consistent relative performance gains under varying sensing noise and write-error probabilities.

In the first experiment, the value of $H_{min}$ is varied to determine the corresponding optimized detection threshold $R_{th}$ and bit-one probability $P_{1}$ as functions of the normalized sensing noise $\sigma_{0}/\mu_{0}$. The resulting optimized operating points $\left(P_{1}^{*},R_{th}^{*}\right)\;$ obtained from the analytical joint optimization are summarized in Table 1. These values characterize the design targets of the proposed scheme. However, overall system reliability also depends on coding, decoding, and dynamic channel behavior. Therefore, in the following experiments, full behavioral simulations are conducted using the optimized parameters as fixed design inputs.

As shown in Table 1, increasing $H_{min}$ forces the optimized bit distribution to approach a uniform distribution, causing $P_{1}^{*}$ to increases monotonically toward 0.5. Meanwhile, larger sensing noise $\sigma_{0}/\mu_{0}$ shifts the optimal detection threshold upward, reflecting the need for more conservative sensing under severe resistance variation.

In the behavioral BER evaluation, extremely small entropy constraints (e.g., $H_{min}=0.5$ or 0.6) are not considered. Although such settings can achieve very low BER by strongly biasing the codeword distribution, they require excessively long codewords to satisfy the entropy constraint while embedding short user data, resulting in an impractically low coding rate. This makes such configurations unsuitable for sensor-oriented systems where throughput and storage efficiency are critical. Accordingly, we focus on moderate-to-high entropy settings, namely $H_{min}=0.8$, 0.95, and 0.99, which offer a better balance between reliability and coding efficiency.

For each case, the optimized $P_{1}^{*}$ and $R_{th}^{*}$ obtained from the analytical framework are fixed and used in system-level simulations. The parameter m mainly controls the stability of the codeword weight distribution rather than the target bit probability, which is primarily determined by the inversion threshold. Following [28], we fix *m* = 3 to balance stability and redundancy while maintaining low encoder complexity. This choice ensures that the BER results primarily reflect the impact of the entropy constraint and the optimized detection threshold.

As shown in Figure 6, the BER of all configurations increases monotonically with increasing sensing noise $\sigma_{0}/\mu_{0}$. Among the proposed settings, $H_{min}=0.8$ achieves the lowest BER, whereas $H_{min}=0.99$ yields the highest BER due to its near-uniform bit distribution. This result confirms the inherent trade-off between information content and reliability: lower entropy enables stronger bias toward the more reliable resistance state and consequently reduces BER. Compared with existing mapping-based and ECC-based schemes, the proposed TMPC framework with $H_{min}=0.8$ and 0.9 consistently outperforms 7/9 mapping scheme [7] and Bose–Chaudhuri–Hocquenghem (BCH) coding [25], and achieves comparable or superior performance relative to the 54/63 coding scheme [28] in low-to-moderate noise regions. In the proposed TMPC scheme, the target bit-one probability $P_{1}^{*}$ only determines the inversion threshold *τ* and thus controls the composition of the codeword, but it does not change the number of valid codewords. Since the conditional inversion is a one-to-one mapping, the size of the codebook remains unchanged, and therefore the coding rate is independent of $P_{1}^{*}$ and *τ*. The coding rate is solely determined by the underlying linear code parameters and the number of appended zero bits. Following this principle and for fair comparison with prior work, we adopt the same coding rate of 54/63 as in [28] throughout our experiments.

At $\sigma_{0}/\mu_{0}=10$, the proposed TMPC with $H_{min}=0.8$ achieves a BER of approximately $4\times 10^{-6}$, whereas the 54/63 scheme yields $3\times 10^{-5}$. This corresponds to a BER reduction of approximately 86.67%.

At $\sigma_{0}/\mu_{0}=14$, the proposed scheme achieves nearly 0.62 orders of magnitude lower BER than the 7/9 mapping scheme. These results confirm the substantial reliability improvement provided by the joint optimization framework.

The threshold-only optimization (with $P_{0}=0.5$) provides moderate BER improvement over the fixed design. However, the proposed joint optimization consistently achieves lower BER, confirming that additional gain arises from adapting the bit distribution to channel asymmetry.

The entropy constraint $H_{min}$ determines the allowable deviation from equiprobable bit distribution. As $H_{min}$ decreases, the optimizer is allowed to bias the codeword composition toward the more reliable resistance state, resulting in lower BER. This trend is clearly observed in Figure 6, where $H_{min}=0.8$ achieves the lowest BER among the tested configurations.

Importantly, the coding rate remains unchanged at 54/63 for all entropy settings. This is because the proposed TMPC employs a one-to-one conditional inversion that preserves the number of valid codewords. Therefore, $H_{min}$ affects the reliability performance but does not directly alter the coding rate.

Finally, we set $\sigma_{0}/\mu_{0}=\sigma_{1}/\mu_{1}=9.5\%$ and vary the write error probability $P_{1}$ (corresponding to the 0 → 1 transition) during the write process [28]. The resulting BER performance is shown in Figure 7.

To further evaluate robustness, we additionally include a mismatch scenario for $H_{min}=0.8$, where the optimal $\left(P_{1}^{*},R_{th}^{*}\right)$ obtained at the nominal $P_{1}$ and $\sigma_{0}/\mu_{0}=\sigma_{1}/\mu_{1}=9\%$ are kept fixed while the actual write error rate $P_{1}$ varies. As shown in Figure 7, the BER degradation remains smooth and limited. The gap between the fixed-design and re-optimized curves is small across the tested range, indicating that the proposed TMPC framework exhibits strong local robustness against channel drift.

Since the proposed TMPC scheme reuses the same encoder and syndrome-based decoder architecture as in [28], its online computational complexity is essentially identical to that of the reference design. The additional operations introduced by TMPC are limited to offline optimization of the detection threshold and target bit distribution, which is performed only once for a given channel condition and does not affect real-time encoding or decoding. Consequently, the proposed method achieves substantial BER improvement without increasing online computational complexity.

The conventional threshold-only scheme optimizes only the detection threshold, resulting in a complexity of *O*(*T*), where *T* denotes the number of iterations required by the numerical solver. In contrast, the proposed joint optimization performs alternating updates of both threshold and bit distribution, leading to a complexity of *O*(*IT*), where *I* denotes the number of outer iterations.

In practice, *I* is typically smaller than 10 and *T* is on the order of log(1/ε). Therefore, the proposed method introduces only a small additional offline computational cost. Importantly, both schemes have identical online encoding and decoding complexity, as the coding structure remains unchanged.

## 5. Conclusions

This paper proposed a TMPC framework for asymmetric STT-MRAM channels based on a cascaded write–read statistical model. Unlike conventional approaches that independently optimize detection thresholds or employ fixed bit distributions, the proposed method formulates BER minimization as a joint optimization problem over both detection thresholds and codeword bit distributions under an entropy constraint.

Simulation results demonstrate that the proposed joint optimization strategy consistently achieves lower BERs compared with conventional sparse coding and threshold-only schemes across varying sensing noise levels and write asymmetry conditions. In particular, the performance gain remains stable under parameter variations, confirming the robustness of the proposed framework.

In terms of computational complexity, the additional cost introduced by joint optimization is limited to offline parameter estimation with complexity *O*(*IT*), where *I* denotes the number of outer iterations and *T* corresponds to the numerical solver iterations. The online encoding and decoding complexity remains identical to that of the underlying linear block code.

While the present work focuses on analytical joint optimization under a cascaded memory-channel model, future extensions may explore data-driven or learning-based approaches for adaptive parameter tuning under time-varying device conditions. In other domains, such as wireless resource allocation, deep reinforcement learning has shown effectiveness in handling complex dynamics with reduced model dependence [35]. Investigating similar adaptive strategies for memory-channel optimization remains an interesting direction for future research.

## Figures and Tables

**Table 1:** Optimized bit probability $P_{1}^{*}$ and detection threshold $R_{th}^{*}$ obtained from the proposed joint optimization under different entropy constraints and sensing noise levels.

	$H_{min}$	0.5	0.6	0.7	0.8	0.9	0.95	0.98	0.99
$\sigma_{0}/\mu_{0}$		$\left(P_{1}^{*},R_{th}^{*}\right)$	$\left(P_{1}^{*},R_{th}^{*}\right)$	$\left(P_{1}^{*},R_{th}^{*}\right)$	$\left(P_{1}^{*},R_{th}^{*}\right)$	$\left(P_{1}^{*},R_{th}^{*}\right)$	$\left(P_{1}^{*},R_{th}^{*}\right)$	$\left(P_{1}^{*},R_{th}^{*}\right)$	$\left(P_{1}^{*},R_{th}^{*}\right)$
8		(0.11, 1.36)	(0.15, 1.36)	(0.18, 1.36)	(0.24, 1.35)	(0.31, 1.35)	(0.36, 1.34)	(0.41, 1.34)	(0.44, 1.34)
9		(0.11, 1.37)	(0.15, 1.37)	(0.18, 1.36)	(0.24, 1.36)	(0.31, 1.35)	(0.36, 1.35)	(0.41, 1.34)	(0.44, 1.34)
10		(0.11, 1.38)	(0.15, 1.38)	(0.18, 1.37)	(0.24, 1.36)	(0.31, 1.36)	(0.36, 1.35)	(0.41, 1.35)	(0.44, 1.35)
11		(0.11, 1.39)	(0.15, 1.39)	(0.18, 1.38)	(0.24, 1.37)	(0.31, 1.36)	(0.36, 1.36)	(0.41, 1.35)	(0.44, 1.35)
12		(0.11, 1.41)	(0.15, 1.4)	(0.18, 1.39)	(0.24, 1.38)	(0.31, 1.37)	(0.36, 1.36)	(0.41, 1.36)	(0.44, 1.35)
13		(0.11, 1.42)	(0.15, 1.41)	(0.18, 1.4)	(0.24, 1.39)	(0.31, 1.38)	(0.36, 1.37)	(0.41, 1.36)	(0.44, 1.36)
14		(0.11, 1.43)	(0.15, 1.42)	(0.18, 1.41)	(0.24, 1.40)	(0.31, 1.38)	(0.36, 1.37)	(0.41, 1.37)	(0.44, 1.36)
15		(0.11, 1.44)	(0.15, 1.43)	(0.18, 1.42)	(0.24, 1.41)	(0.31, 1.39)	(0.36, 1.38)	(0.41, 1.37)	(0.44, 1.37)
16		(0.11, 1.46)	(0.15, 1.44)	(0.18, 1.43)	(0.24, 1.42)	(0.31, 1.4)	(0.36, 1.39)	(0.41, 1.38)	(0.44, 1.37)

## Data Availability

The data presented in this study are available in article.
